# Short‐term effect of elevated CO_2_ concentration (0.5%) on mitochondria in diploid and tetraploid black locust (*Robinia pseudoacacia* L.)

**DOI:** 10.1002/ece3.3046

**Published:** 2017-05-22

**Authors:** Fuling Xu, Mingquan Jiang, Fanjuan Meng

**Affiliations:** ^1^ College of Life ScienceNortheast Forestry UniversityHarbinChina; ^2^ Jilin Province Product Quality Supervision and Inspection InstituteChangchunChina

**Keywords:** 0.5% CO_2_, mitochondria, *Robinia pseudoacacia* L.

## Abstract

Recent increases in atmospheric CO_2_ concentration have affected the growth and physiology of plants. In this study, plants were grown with 0.5% CO_2_ for 0, 3, and 6 days. The anatomy, fluorescence intensity of H_2_O_2_, respiration rate, and antioxidant activities of the mitochondria were analyzed in diploid (2×) and tetraploid (4×) black locust (*Robinia pseudoacacia* L.). Exposure to 0.5% CO_2_ resulted in clear structural alterations and stomatal closure in the mitochondria. Reduced membrane integrity and increased structural damage were observed in 2× plants at 6 days. However, after 0.5% CO_2_ treatment, little structural damage was observed in 4× plants. Under severe stress, H_2_O_2_ and malondialdehyde were dramatically induced in both 2× and 4× plants. Proline remains unchanged at an elevated CO_2_ concentration in 4× plants. Moreover, the total respiration and alternative respiration rates decreased in both 2× and 4× plants. In contrast, the cytochrome pathway showed no decrease in 2× plants and even increased slightly in 4× plants. The antioxidant enzymes and nonenzymatic antioxidants, which are related to the ascorbate–glutathione pathway, were inhibited following CO_2_ exposure. These analyses indicated that 4× and 2× plants were damaged by 0.5% CO_2_ but the former were more resistant than the latter, and this may be due to increases in antioxidant enzymes and nonenzymatic antioxidants and stabilized membrane structure.

## 1 INTRODUCTION

Currently, due to anthropogenic activity, CO_2_ concentrations have increased since the pre‐industrial period from 280 to 401.85 μmol/mol (NOAA‐ESRL; 2014, M. Lambreva 2005). Many reports have estimated that this value will increase to 1000 ppm by the end of the century if no corrective measures are undertaken to constrain emissions (IPCC, 2014). In general, elevated CO_2_ concentrations (EC) directly affect crop yield in many species by increasing carboxylation efficiency, enhancing intercellular CO_2_ concentration, and reducing photorespiration (Bowes, 2003; Sharkey, 1985; Stiling et al., 2013). Furthermore, C_3_ and C_4_ plants respond differently to CO_2_. According to early reports, elevated CO_2_ concentrations will most probably favor C_3_ plant types than C_4_ because currently, the concentration of CO_2_ in the atmosphere is inadequate to saturate the ribulose‐1,5‐ bisphosphate carboxylase (RuBisCO) enzyme that drives photosynthesis in C_3_ plants (Taiz & Zeiger, 1991). In comparison, C_4_‐type plants are likely to respond less to elevated CO_2_ levels as they possess an innate concentrating mechanism that increases CO_2_ level at the site of RuBisCO to 2,000 ppm. Moreover, studies have indicated that EC affects rise of tiller number, net photosynthetic rate, and morphology, as well as yield enhancement (Hasegawa et al., 2013; Liu et al., 2008; Shimono et al., 2009). To date, variability in plant respiration has been observed and the underlying mechanism has been elucidated (Hu et al., 2006). While plant growth, development, and function in increased CO_2_ concentrations have been extensively researched (Zinta et al., 2014), the effects of elevated CO_2_ on the ultrastructure and function of mitochondria in polyploid plants are relatively unknown.

Tetraploid black locust (*Robinia pseudoacacia* L.) (TBL) is native to Korea. This species is cultivated for its wood throughout the world (Isely & Peabody, 1985). Its leaves are rich in many types of vitamins and minerals, which can be used for the food industry. Moreover, TBL can tolerate abiotic stresses, such as drought, salt, and low temperature (Joshi, Bourges‐Sévenier, Russell, & Mo, 2012). Its bark and roots can be used for drug development and disease treatment due to its abundance of flavonoids (Garlock, Yi, Balan, & Dale, 2012). Because of its rapid growth and pleasant fragrance, TBL has been cultivated as a useful component of secondary forests in gardens and on roads. However, the response of TBL to high concentrations of CO_2_ has not been elucidated.

Many polyploid plants have a higher tolerance to environmental stresses than their diploid counterparts (Meng, Pang, Huang, Liu, & Wang, 2012). Conversely, research has shown that the volume of organelles in polyploid plants is greater than that in their corresponding diploid relatives. Nevertheless, mitochondria are ultimately responsible for oxidative phosphorylation in metabolic processes (Plaxton & Podestá, 2006) and are sensitive to various abiotic stresses (Halliwell & Gutteridge, 1981). Mitochondria are the primary cellular organelles that respond to elevated CO_2_ levels, which has been the topic of extensive research. While the acute response of plants to elevated CO_2_ has been studied, the precise mechanism by which EC affects mitochondria in polyploid woody plants is presently unknown. In particular, understanding the basis for this variation in polyploid woody plants exposed to rising CO_2_ concentrations is important for further selection and development of elevated [CO_2_]‐responsive crop lines.

CO_2_ as a abiotic factor can affect plant growth and development. Previous studies have suggested that changes in morphology and photosynthesis may be related to high CO_2_. However, little or no work has been performed to investigate black locust responses to high CO_2_. In this study, the effect of elevated concentrations of CO_2_ on the anatomy, respiration, and antioxidant activity of diploid (2×) and tetraploid (4×) plants was studied after the plants were exposed to 0.5% CO_2_ for 0 (up) and 6 (down) days. Based on this analysis, we increased our understanding of the mechanism underlying the CO_2_ response and tolerance of plants to environmental stress.

## 2 MATERIALS AND METHODS

### 2.1 Plant growth

All materials were obtained from the Beijing Forest University and were introduced directly from Korea to China. Diploid (2×) and tetraploid (4×) black locust (*Robinia pseudoacacia* L.) plants were cultivated in plastic pots (10 cm^2^). When the plants were 2 months of age, they were treated with 0.5% CO_2_ in high‐performance CO_2_‐controlled growth chambers (Huanghua Faithful Instrument Co., Ltd. Hebei, China) (0.5%, control and treated) (light/dark 16 hr/8 hr, at temperatures of 35/25°C, 50/60% humidity and 250 μmol photons/m^2^/s light). After 0, 3, and 6 days of exposure to 0.5% CO_2_ treatment, leaves were randomly harvested and stored at −80°C.

### 2.2 Stomatal aperture and chloroplast ultrastructure

Fresh leaf sections (1 cm^2^) were immediately fixed in 3% glutaraldehyde in 0.1 M phosphate buffer at 4°C for 2 hr and thoroughly washed in cacodylate buffer (0.1 M, pH 6.8) twice, with 10‐min intervals between each washing. They were then dehydrated in a graded ethanol series (30%, 50%, 70%, 80%, 90%, 90.5%, and 100%) with 10 min each time, and the 100% ethanol wash was repeated twice. After dehydration, the samples were further dried in acetone and embedded in an Epon–Araldite mixture. For scanning electron microscopy (SEM), the samples were pasted to copper stubs with colloidal silver and were sprayed with 50 nm gold. Then, the samples were observed and photographed using a scanning electron microscope (JSM‐5310LV, Japan). The stomatal aperture size was calculated using ImageJ 1.4.7 software (ImageJ, Bethesda, MD, USA).

### 2.3 Determination of H_2_O_2_ in guard cells

Detection of H_2_O_2_ in guard cells was performed as previously described by Comai (2005). H_2_O_2_ generation in the stomata was assessed with H_2_DCF (2, 7‐dichlorodihydrofluorescein diacetate), a specific fluorescence probe for H_2_O_2_. The young leaves were harvested after 0, 3, and 6 days of 0.5% CO_2_ treatments from both 2× and 4× plants. The young epidermal strips were incubated in 10 mM MES–KCL buffer (pH 7.2). Then, 50 μM H_2_DCF was added at room temperature and incubated for 20 min in the dark. The leaves were rinsed with MES buffer twice to remove the additional fluorescence detector. The fluorescence of the H_2_O_2_ probe was measured with an Axioskop 2 plus microscope (Zeiss) (excitation wavelengths of 488 nm).

### 2.4 Respiration measurements

Leaf respiration was measured at room temperature using a Clark oxygen electrode (Hansatech, England) that was inserted into a 2‐ml cuvette on a magnetic stirrer. The data were collected by a computer. The leaf tissues (0.1 g) were sliced (1 mm^2^) and submerged in incubation buffer (2 mM CaCl_2_, 10 mM HEPES, and 10 mM MES, pH 7.2). The slices were continuously stirred to dissolve the oxygen. Alternative respiration (alt) was sensitive to SHAM, and cytochrome respiration (cyt) was sensitive to NaN_3_. To distinguish between alt respiration and the cyt respiration, we added 5 mM SHAM and 0.1 mM NaN_3_ to the suspension, respectively. The rate of respiration was surveyed at 5 min in the presence of inhibitors. We then calculated the percentage inhibition of the total respiration rates.

### 2.5 Isolation of mitochondria

The plant leaves (1.5 g) were harvested, and the mitochondria were isolated using an extraction buffer that contained 20 mM EDTA, 50 mM HEPES/KOH (pH 7.5), 30 mM Na ascorbate, 5 mM hexanoic acid, 10 mM NaCl, 0.3 M sucrose, 1% (w/v) PVP, 10 mM β‐mercaptoethanol, and 0.3% BSA (w/v). After filtration through six layers of Miracloth, the crude material was filtered and collected. Then, the homogenate was centrifuged at 4,000 r/min for 10 min. The supernatant was centrifuged at 10,000 r/min for 10 min. The pellet was resuspended in washing buffer 20 mM HEPES/KOH (pH 7.8), 330 mM sorbitol, 10 mM NaCl, 2 mM EDTA, and 5 mM Na ascorbate] and centrifuged for 20 min at 4000 r/min. Then, the supernatant was centrifuged at 10,000 r/min for 10 min. The intact mitochondria were collected, washed, and centrifuged at 12,000 r/min for 20 min in PBS.

### 2.6 Antioxidant enzyme activity in mitochondria

Ascorbate peroxidase (EC 1.11.1.11) was examined at 290 nm according to the method of Nakano and Asada (1980). The reaction mixture contained 5 mM ascorbic acid (AsA), 50 μl enzyme extract, 0.2 M phosphate buffer (pH 6.8), and 2 mM H_2_O_2_. The MDHAR activity (monodehydroascorbate reductase 1.6.5.4) was determined spectrophotometrically by monitoring the change in 340 nm. The reaction mixture contained 0.1 mM NADH, 0.1 mM AsA, and 0.55 U AAO, 50 μl enzyme extract, and 0.2 M phosphate buffer (pH 6.8). DHAR (EC 1.8.5.1) activity was measured following the method of Dalton and Evans (1986) at 265 nm. The glutathione reductase activity was assayed at 340 nm following Madamanchi and Alscher (1991). The reaction mixture contained 10 mM GSSG, 2 mM NADPH, 50 μl enzyme extract, and 0.2 M phosphate buffer (pH 6.8).

### 2.7 Analysis of H_2_O_2_, MDA, and proline

H_2_O_2_ was analyzed according to Sergiev et al. (1997). The absorbance of the supernatant was measured at 390 nm, and the H_2_O_2_ concentration was obtained using a standard curve. For measurement of malondialdehyde (MDA) content, 1.5 g fresh leaf was homogenized with 0.1% trichloroacetic acid (TCA). After centrifugation at 12,000 r/min for 10 min, 1 ml of the supernatant was collected and mixed with 2 ml 0.5% thiobarbituric acid (TBA). Then, the mixtures were heated in boiling water (100°C) for 30 min. The homogenate was centrifuged at 12,000 r/min for 5 min. The absorbance changes at 450, 532, and 600 nm were monitored at 25°C. Proline content was measured following the method of Kong et al. (2014), with minor modifications. The leaves (1.5 g) were extracted in 3% sulfosalicylic acid in a mortar and pestle at 4°C. After incubation at 100°C for 10 min, 1.5 ml of ninhydrin reagent (2.5% ninhydrin, 60% glacial acetic acid, and 40% 6 M phosphoric acid) and 1 ml of glacial acetic acid were added to 1 ml of the leaf extract at room temperature. The mixture was heated again in boiling water (100°C) for 30 min. Then, 3 ml of toluene was added, and the sample was incubated on an ice bath for 1 hr. The absorbance change was monitored at 520 nm.

### 2.8 Measurements of nonenzymatic antioxidants

Total AsA content (AsA+DHA) was determined using a modified protocol from Law, Charles, and Halliwell (1983). Briefly, 1.5 g of leaf sample was homogenized with 0.5% sulfosalicylic acid, and the supernatants were obtained by centrifugation for 20 min (12,000 r/min; 4°C). Then, 10 mM DTT and 0.5% N‐ethylmaleimide were added to 0.1 ml extract. After the sample was mixed for one minute, 0.7 ml double‐distilled water, 4% α′‐dipyridyl in 70% ethanol, 10% trichloroacetic acid and 44% phosphoric acid were successively added to the mixture. Then, the mixture was heated to 40°C for 40 min. Then, 3% FeCl_3_ was added. The absorbance was measured at 525 nm. AsA content was determined as total AsA, with the exception of 10 mM DTT and 10 mM N‐ethylmaleimide.

The total content of reduced glutathione (GSH+GSSG) was measured as described previously Ellman (1959). Briefly, 100 μl of the extract was incubated with 100 mM PBS, 0.6 mM DTNB, and 2 mM NADPH for 10 min at 25°C. The reduced glutathione content (GSH) was measured at 412 nm as total reduced glutathione, with the exception of 2 mM NADPH.

## 3 RESULTS

### 3.1 Mitochondrial ultrastructure

**Figure 1:**
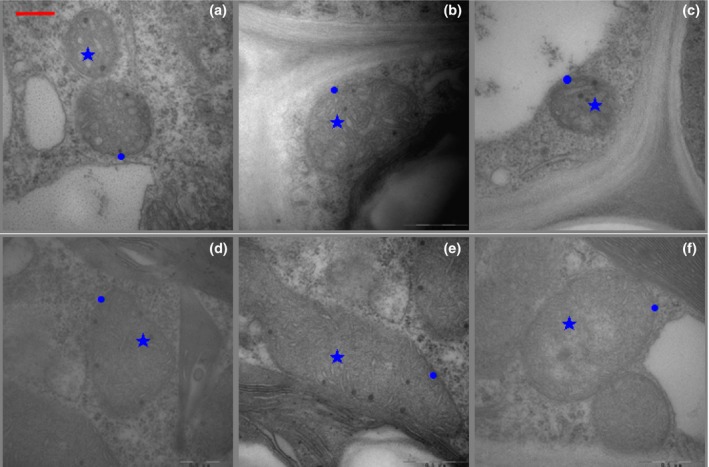
Transmission electron micrographs of mitochondria in 2× (a–c) and 4× (d–f) plants that were grown under 0.5% CO_2_ conditions at 0, 3, and 6 days. 2×, diploid black locust; 4×, tetraploid black locust; Five‐pointed star (★), mitochondrial cristae; ball (●), membranes of mitochondria. Bar = 5 μm

The 0.5% CO_2_ treatments induced notable disturbances in mitochondrial morphology. The mitochondrial ultrastructure of the 2× and 4× plants is shown in Figure 1. The oval or rounded mitochondrial ultrastructure was elongated, sometimes branched, or dumbbell shaped (Figure 1a,d); however, mitochondrial membranes and cristae after 3 days of 0.5% CO_2_ displayed minor injuries in the 2× plants (Figure 1b) compared with those of the 4× plants (Figure 1e). In the 2× plants, another set of mitochondria had a low number of cristae and large electron‐transparent areas in the matrix (Figure 1c) after 6 days of 0.5% CO_2_. Conversely, after the 0.5% CO_2_ stress treatments for 6 d, mitochondria in the 4× plant leaf mesophyll cells showed less damage (Figure 1f) than those of the 2× plants.

### 3.2 Stomata morphology

**Figure 2:**
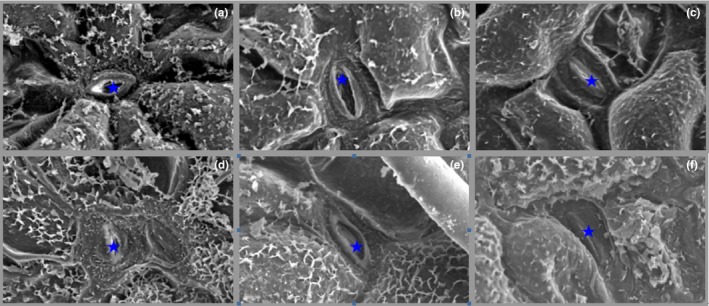
Transmission electron micrographs of stomata in 2× (a–c) and 4× (d–f) plants that were grown in 0.5% CO_2_ conditions at 0, 3, and 6 days. Five‐pointed star (★), stomata. Bar = 5 μm

**Figure 3:**
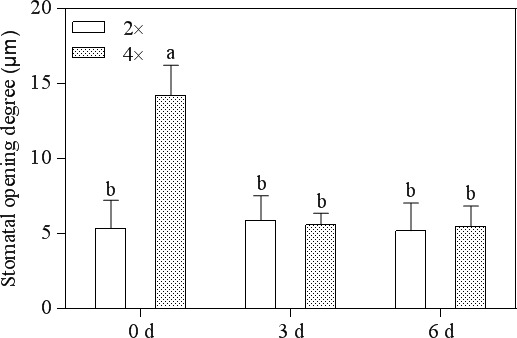
Effect of 0.5% CO_2_ on stomatal opening degree in 2× and 4× (*p *<* *.05)

Figure 2a,d shows a typical SEM image of the 2× and 4× plant stomata structure. Stomatal openings in the leaves of 2× plants that were exposed to CO_2_ for 3 days were fewer than those of the control (Figures 2b, 3). At the same time, exposure to the gaseous CO_2_ for 6 days resulted in total closure of the 2× plant leaves (Figure 2c). Interestingly, in the 4× plants, 0.5% CO_2_ induced the complete closure of guard cells after 3 days (Figure 2e,f). Figure 3 shows significant changes in the degree of stomatal opening in the 4× plants. In contrast, there was no significant change in the 2× plants.

### 3.3 Fluorescence intensity of H_2_O_2_

**Figure 4:**
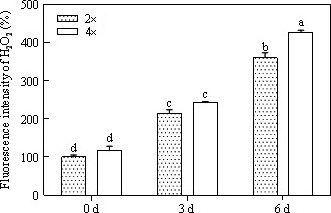
Scanning fluorescence intensity of H_2_O_2_ in mitochondria of leaves in 2× and 4× plants that were grown in 0.5% CO_2_ conditions for 0, 3, and 6 days (*p *<* *.05). a, b, c and d shows the results of the significance analysis

As expected, after 0.5% CO_2_ exposure, accumulation of H_2_O_2_ in the 4× plants was greater than that in the controls (Figure 4). During the treatment, H_2_O_2_ levels increased gradually at 3 and 6 days in the 2× plants (Figure 4). The relative fluorescence intensities of the 2× plants after 6 days of CO_2_ stress was approximately 3.6‐fold that of the control (Figure 4). Moreover, accumulation of H_2_O_2_ was significantly increased after 3 days of exposure to 0.5% CO_2_ in 4× plants compared to that of 2× plants (Figure 4).

### 3.4 Changes in H_2_O_2_, MDA, and proline

**Figure 5:**
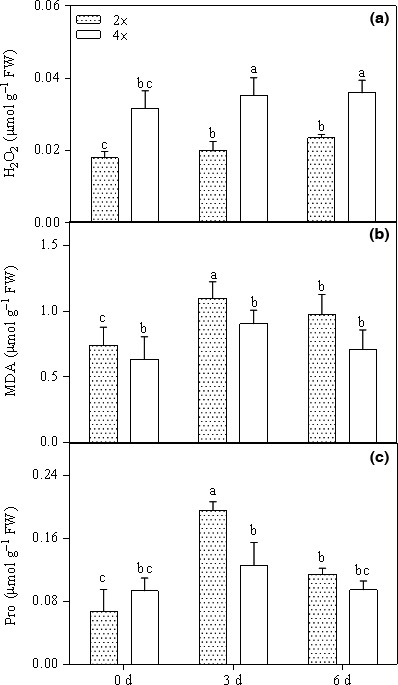
Hydrogen peroxide (a), malondialdehyde (b), and proline (c) contents of the leaves in mitochondria of leaves in 2× and 4× plants that were grown in 0.5% CO_2_ conditions for 0, 3, and 6 days (*p *<* *.05). a, b, c and d shows the results of the significance analysis

MDA was induced by 48.24% and 31.4% after 3 and 6 days of stress, respectively (Figure 5b). Proline content in the 2× plants was significantly enhanced by 70.2% after CO_2_ stress for 6 days (Figure 5c); however, these values were similar in the 4× plants between the control and treatment (Figure 5b,c). Moreover, the accumulation of H_2_O_2_ in 2× plants after 6 days increased to 31.1% that of the CK (Figure 5a). Furthermore, H_2_O_2_ content in 4× plants increased by 13.79% under severe stress (Figure 5a).

### 3.5 Leaf respiration rate

**Figure 6:**
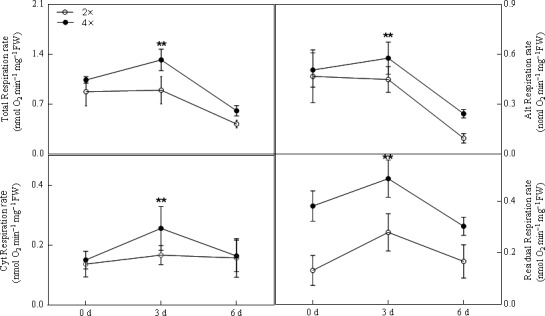
Leaves respiratory responses following various days under 0.5% CO_2_ conditions in 2× and 4× plants at 0, 3, and 6 days. Asterisks indicate significant differences on each day after treatment (**p < .01). (a) Total respiration of 2× and 4×plants at 0, 3, and 6 days, respectively; (b) Alt (alternative) respiration of 2× and 4×plants at 0, 3, and 6 days, respectively; (c) Cyt (cytochrome) respiration of 2× and 4×plants at 0, 3, and 6 days, respectively; (d) Residual respiration of 2× and 4×plants at 0, 3, and 6 days, respectively

To investigate the effects of high concentrations of CO_2_ on plant respiration, we measured leaf respiration at 0, 3, and 6 days. As shown in Figure 6a, total respiration of the plants was temporarily increased at 3 days under elevated CO_2_ compared to that at 6 days in 4× plants. In contrast, this ratio was decreased in 2× black locust plants under stress conditions (Figure 6a). The total respiration decreased to 51.9% and 41.6% in 2× and 4× plants, respectively (Figure 6a). Conversely, the alt respiration of 4× plants was slightly induced initially and clearly decreased at 6 days; however, 2× plants that were grown under CO_2_ stress conditions showed a significant decrease in alt respiration rate from 0 to 6 days (Figure 6b). The responses of plant cyt respiration to CO_2_ were also explored (Figure 6c). There were no significant changes in 2× plants compared with those in 4× plants, which showed a substantial alteration. The change in trend of residual respiration was similar to that of alt respiration in black locust leaves (Figure 6d).

### 3.6 The AsA/GSH antioxidant defense system

**Figure 7:**
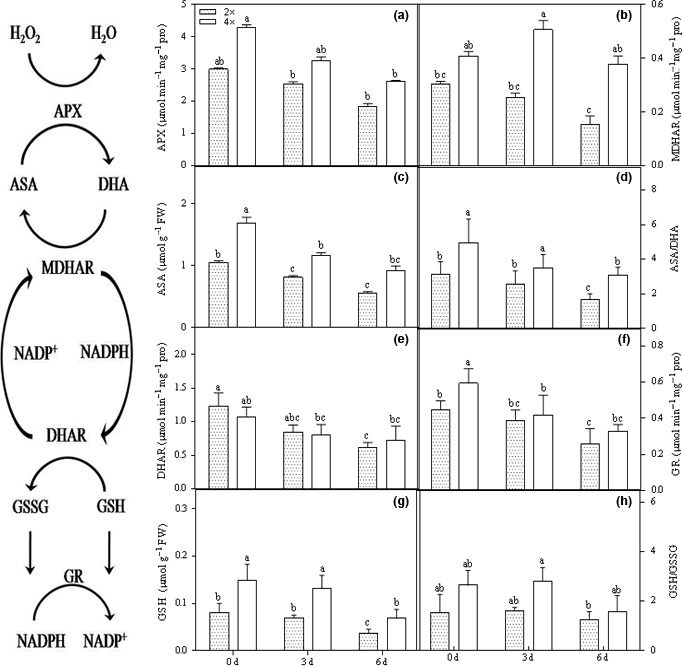
Responses of ASA/GSH defense. Responses of the ascorbate/glutathione (ASA/GSH) defense in 2× and 4× plants under CO_2_ conditions. Line graphs represent, (a) ascorbate peroxidase (APX) activity; (b) monodehydroascorbate reductase (MDHAR) activity; (c) reduced ascorbate (ASA) levels; (d) ascorbate redox status (ASA/DHA); (e) dehydroascorbate reductase (DHAR) activity; (f) glutathione reductase (GR) activity; (g) reduced glutathione levels (GSH); (h) glutathione redox status (GSH/GSSG) (*p *<* *.05) . a, b, c and d shows the results of the significance analysis

DHAR activity was significantly reduced at the beginning of stress in both the 2× and 4× plants (Figure 7e). AsA levels significantly decreased under 0.5% CO_2_ conditions in both the 2× and 4× plants (Figure 7c). Interestingly, changes in GSH were notable at 6 days after 0.5% CO_2_ treatment (Figure 7g). The APX and GR activities decreased moderately in 2× plants under stress conditions but were not significantly changed in 4× plants (Figure 7a,f). Increased CO_2_ reduced the APX activities in 2× and 4× plants by 38.4% and 39.1%, respectively (Figure 7a). Furthermore, the GR activity gradually declined by 42.0% and 44.5% in 2× and 4× plants, respectively (Figure 7f). MDHAR was not significantly altered by elevated CO_2_ in 4× plants, but it was significantly decreased in 2× plants (Figure 7b). In addition, stress significantly impacted GR, APX, and MDHAR activity in 4× plants. Elevated CO_2_ had little effect on DHAR in 4× plants, but it was notably reduced in 2× plants.

## 4 DISCUSSION

Due to anthropogenic carbon emissions and ecosystem processes, excessive CO_2_ has been released into the atmosphere. This is predominantly due to cement production, fossil fuel burning, and land‐use change (Leakey et al., 2009) resulting in climate change and dramatic increases in the CO_2_ concentration. The changing CO_2_ concentration has a vital role in plant growth and development. Many reports have shown that TBL is highly tolerant to environmental stresses (Li et al., 2009; Podda et al., 2013; Yuan, Liu, Fang, Yang, & Mu, 2009). Moreover, polyploidy induction was used as an important method to assess plant physiological mechanisms under severe conditions (Comai, 2005; Woode et al., 2009). To date, few studies have examined the effects of a high concentration of CO_2_ on TBL. Thus, the plant responses to 0.5% CO_2_ were investigated in this study.

Generally, plants exposed to high concentrations of CO_2_ generate excess excitation pressure, which can supply electrons in excess of that required for CO_2_ fixation (Asada, 1999; Murchie & Niyogi, 2011). This results in the formation and accumulation of reactive oxygen species (H_2_O_2_, ·OH and O_2_^·‐^) (Tausz‐Posch et al., 2013). In the current study, H_2_O_2_ content was detected in both 2× and 4× plants and was shown to significantly increase at 3 days under stress conditions (Figure 4). At the same time, a higher H_2_O_2_ content was observed in the leaves at 4× plants (Figure 4) compared that in the leaves of 2× plants (Figure 4) in 0.5% CO_2_ conditions. The hormone abscisic acid (ABA) is induced by stress and activates NADPH oxidase to generate H_2_O_2_ in guard cells (Sridharamurthy et al., 2014). In particular, H_2_O_2_ can control the activity of outward/inward‐rectifying K^+^ channels, enhance guard cell Ca^2+^ concentrations, and induce cytosolic alkalinization, which results in stomatal closure through trigger water efflux (Brearley, Venis, & Blatt, 1997; Felle & Hanstein, 2002; Raschke, Shabahang, & Wolf, 2003). Stomata on the leaf surface continuously regulate gas exchange by a sophisticated mechanism in response to environmental changes. In this study, 2× plant stomatal closure was observed at 6 days and 4× plant closure was observed after 3 days (Figure 2c,e). Stomatal closure may represent a protective response to abiotic stress. We found that 4× plants responded more rapidly to the stress than did 2× plants, which can decrease the CO_2_ flowing into the leaves (Figure 3). The degree of stomatal opening significantly decreased in 4× plants at 3 days compared to that of 2× plants (Figure 3). Similar responses have been reported for adaptation to other stress conditions, including drought, cold, salinity, and carbon monoxide (He & He, 2014; Himmelbach, 1998; Hubbard, Nishimura, Hitomi, Getzoff, & Schroeder, 2010).

Mitochondria act as sensors, initiate stress responses in plant, and are major producers of ROS (Nomura et al., 2012; Suzuki, Koussevitzky, Mittler, & Miller, 2011; Vanlerberghe, 2013). Under abiotic stress conditions, ROS were generated and accumulated in the guard cells, inducing both cellular damage and protective responses (Wu et al., 2013). In this report, the 0.5% CO_2_ stress treatments induced notable disturbances in mitochondrial structure (Figure 1). Ultrastructural observations of the mitochondria showed that the cristae in 2× plants were reduced under severe stress and appeared empty, similar to vacuoles (Figure 1b,c), compared with those of the control (Figure 1a). Additionally, nonenzymatic activity has an important role in scavenging ROS in all plant cells. MDA and proline are the most important molecules that regulate and adapt to environmental stress in plants and were significantly increased in 2× plants (Figure 5b,c). In parallel with the H_2_O_2_ accumulation, MDA, which is an indicator of oxidative damage to the membranes, and mitochondrial membranes showed alterations in several regions of the 2× plants after 6 days at CO_2_ treatment (Figure 1c). In contrast to the 2× plants, the 4× plants showed fewer mitochondrial changes during stress (Figure 1e,f), which indicates that 4× plants are more resistant to CO_2_ stress than 2× plants. A recent study suggested that changes in mitochondrial morphology are early indicators that cells are affected by ROS (Grigorova et al., 2013).

Vassileva et al. reported that stress can promote mitochondrial inner membrane permeabilization. Plant mitochondria possess a branched electron transport chain (Kühn et al., 2015). The plant inner mitochondria membrane contains both the SHAM‐resistant cyt pathway and the KCN‐resistant alt pathway. Abiotic stress had direct effects on mitochondrial respiration. Previous studies have shown that the respiration rate varies following different environment stresses (Hu et al., 2006). In the present study, 0.5% CO_2_ treatment partly induced stomatal closure in both 2× and 4× plants. Therefore, the rate of total respiration decreased to 51.90% and 41.59% in both 2× and 4× plants, respectively (Figure 6a). A similar trend was observed in the alt respiration rate, which decreased by 79.47% and 51.96% in both 2× and 4× plants, respectively (Figure 6b). Compared to the substantial decrease in the rate of the alt respiration, the cyt pathway showed no decrease in 2× plants and increased slightly in 4× plants (Figure 6c). The alt respiration in 4× plants showed a notable increase compared to that in 2× plants at 3 days. However, the values in both 2× and 4× plants significantly decreased under elevated CO_2_ (0.5%; Figure 6b). According to previous reports, the alt pathway in plant mitochondria can dissipate excessive ROS under abiotic and biotic stresses (Moller, 2001; Siedow & Umbach, 1995). These results indicate that the alt pathway can suppress the production of ROS due to short‐term CO_2_ stress (3 days; Figure 6b). Temporary increases in the alt pathway have been suggested as an adaptive feature of plants exposed to stress (Mcnulty & Cummins, 1987). However, the mitochondrial membrane was damaged by exposure to 0.5% CO_2_ for a longer period of time (6 days) in both 2× and 4× plants (Figure 1c,f). Furthermore, excessive ROS in the mitochondria damage one of the most vital cellular components: proteins/enzymes, which have an important role in plant growth and the antioxidant system. After the treatment, cyt respiration was not affected and was thus less sensitive to CO_2_ than was alt respiration in black locust leaves (Figure 6c).

Under stress conditions, the AsA/GSH pathway is one of the major ROS detoxifying systems in the cytosol. This cycle consists of APX‐, AsA‐, GSH‐, and the AsA/GSH‐regenerating enzymes, including MDHAR, DHAR, and GR (Noctor, 2009) (Figure 7). In this cycle, H_2_O_2_ is reduced to H_2_O by APX, and AsA is oxidized to DHA. Thus, DHA is converted to AsA by DHAR, which catalyzes GSH to GSSH, or the NADPH‐dependent MDHAR enzyme. GR catalyzes the formation of GSH by reducing GSSG. In our experiment, APX and GR were significantly inhibited under stress conditions in 4× plants (Figure 7a,f). Inhibition of APX activity may promote accumulation of H_2_O_2_, which can be determined by MDA content. AsA content was notably reduced after 3 days of stress (Figure 7c). Moreover, the inhibition of GR activity also resulted in a decreased pool of GSH in both 2× and 4× plants (Figure 7f,g). DHAR activity was not changed in 4× plants and decreased in 2× plants following CO_2_ stress (Figure 7e). The GSH/GSSG ratio was increased in 4× plants but did not change in 2× plants (Figure 7h). We also observed that this is a consistent protective response to stress. AsA is a vital nonenzymatic antioxidant in plant cells that eliminates excessive ROS and thus maintains the activity of antioxidant enzymes and stabilizes membrane structure. All results suggested that the 4× plants were more tolerant to CO_2_ than were the 2× plants.

Another study showed that AsA and GSH levels substantially deceased under heat and drought stresses and that the AsA redox status was also higher in elevated CO_2_‐grown plants. Conversely, the antioxidant system was not significantly changed following CO_2_ treatments (730 ppm) (Zinta et al., 2014). In our experiments, the AsA/GSH cycle was inhibited under CO_2_ stress, which was less pronounced under stress conditions. Moreover, in our previous study, 1% and 0.5% CO_2_ were used to treat 2× and 4× plants, respectively, which resulted in the reduction of SOD and GR activity; however, not all antioxidant defense systems responded similarly to the elevated CO_2_ conditions (Zinta et al., 2014). Recent reports have suggested that the inhibition of these antioxidant enzymes may be related to the enhanced oxidative damage in proteins (Aravind & Vara, 2005; Singh, Singh, Kumar, & Prasad, 2015). In conclusion, 4× and 2× plants were damaged by 0.5% CO_2_ but the former was more resistant than the latter, which may be attributable to duplicate gene expression. Ultimately, the mechanisms underlying this difference require further investigation.

## CONFLICT OF INTEREST

The authors declare that they have no competing interests.

## AUTHOR CONTRIBUTIONS

MFJ and XFL conceived and designed the experiments. XFL and JMQ performed the experiments. MFJ, XFL, and JMQ analyzed the data. XFL, MFJ, and JMQ wrote the manuscript; XFL and JMQ provided editorial advice.
